# Temperature change in pulp chamber of primary teeth during curing of coloured compomers: an in vitro study using pulpal blood microcirculation model

**DOI:** 10.7717/peerj.7284

**Published:** 2019-07-08

**Authors:** Ceylan Çağıl Ertuğrul, Ihsan Furkan Ertuğrul

**Affiliations:** 1 Department of Pediatric Dentistry, Pamukkale University, Denizli, Turkey; 2 Department of Endodontics, Pamukkale University, Denizli, Turkey

**Keywords:** Primary teeth, Pulp temperature, Pulp vitality, Coloured compomer, Light curing

## Abstract

**Introduction:**

An increase in the temperature of the pulp chamber occurs during polymerisation of all types of light-curing resin-containing restorative materials, due to both the exothermic reaction of the material and the energy absorbed during the curing process. Increase in temperature of the pulp chamber of primary teeth during the curing process or the thermal conductivity properties of coloured compomers (CCs) have not yet been investigated in detail. The aim of the present study was to investigate the increase in pulpal temperature in primary teeth during curing of CCs.

**Materials and Methods:**

A Class-II cavity was prepared on the extracted primary mandibular second molar. Pulpal microcirculation of the tooth was performed using an experimental mechanism. The study included 15 groups and 10 experiments in each. Seven different CCs: pink, blue, gold, silver, orange, lemon, green, respectively from two different manufacturers (Groups 1–7: Twinky Star; VOCO, Cuxhaven, Germany. Groups 8–14: Nova Rainbow; IMICRYL, Konya, Turkey.) and a tooth-CC (Group 15: Dyract XP; DENTSPLY, Weybridge, UK.) were applied in prepared cavity. In all groups the compomers were light cured for 40 s. Intrapulpal temperature changes (Δ*t*) in 20th and 40th second were recorded. In Group-15 the Δ*t* values in 10th second were also recorded as per the manufacturer’s instructions. The Kruskal–Wallis test and the Mann–Whitney-*U* test were used for statistical analyses.

**Results:**

At the end of 40-s irradiation time, the orange, lemon and green colours of Nova Rainbow resulted in significantly lower Δ*t* values than the same colours of Twinky Star (*p* = 0.0001), and silver, blue, lemon, green, orange, and pink CCs of Nova Rainbow and the blue and silver shades of Twinky Star demonstrated lower Δ*t* values than the reported critical temperature increase (5.5 °C).

**Conclusion:**

An increase in the irradiation time consequently led to an increase in the intrapulpal temperature. Therefore, manufacturers should focus on production of new CCs with shorter polimerization time.

## Introduction

Polyacid modified composite resins, also called compomers, were introduced in 1993 as a new dental material combining the superior physical properties of composite resins and the fluoride releasing property of glass ionomer cements (Nicholson, 2007). Production of the material in different colours commenced in the year 2003 (Croll, Helpin & Donly, 2004) and these glittering and multi-coloured compomers have became attractive for children and therefore result in their cooperation during dental procedures. In addition, enabling children to choose the colour of their own restoration can have a positive effect in overcoming fear of dental procedures and impatience during treatment while also creating enthusiasm regarding maintenance of oral health (Hwang et al., 2007; Yan, 2017). Manufacturers such as Voco Twinky Star and Imicryl Nova Rainbow produce coloured compomers (CCs) that are widely used in paediatric dentistry. A previous study reported that the Twinky Star CC has high compressive strength, good biaxial flexural strength, low rate of wear, good adhesive properties, minimal in vitro cytotoxic effects, and did not cause apparent haemolysis in vivo (Yan, 2017). Despite the advantages of CCs, there is a lack of studies on the possible thermal harmful effects of light-curing process of the material on the primary tooth pulp tissue.

An increase in the temperature of the pulp chamber occurs during polymerisation of all types of light-curing resin-containing restorative materials, due to both the exothermic reaction of the material and the energy absorbed during the curing process (Lloyd, Joshi & McGlynn, 1986; Masutani et al., 1988). Zach & Cohen (1965) reported in their study on Rhesus monkeys that intrapulpal temperature increases of 5.5, 11.1 °C, and above 11.1 °C in monkeys led to 15%, 60% and almost 100% irreversible pulpal damage, respectively (Zach & Cohen, 1965). Besides, it was suggested that duration of temperature increase is an important factor for the irreversible changes in the pulp tissues and the authors reported that 42 °C is a critical temperature when sustained for 1 min duration (Eriksson et al., 1982). However, in a more recent study with human teeth, an increase of 11.2 °C produced no pulpal damage (Baldissara, Catapano & Scotti, 1997).

Temperature increase caused by the light-curing at the cavity wall depends mainly upon the exposure time (McCabe, 1985; Lloyd, Joshi & McGlynn, 1986) and the characteristic of the light source (Bodkin, 1984; Daronch et al., 2007). Furthermore, the shade of the restorative resin, the degree of porosity in the material, the initial resin temperature, and the material thickness may all have an influence on this temperature increase (Durey, Santini & Miletic, 2008). It was recently reported that pigments in darker CCs such as blue shade absorb more light, thus reducing the depth of penetration of the light into the resin material (Jafari, Javadinejad & Mirzakochaki, 2015).

The present study aimed to investigate the increase in intrapulpal temperature in primary molar teeth, at the 20th and 40th second of light curing process of CCs from two different manufacturers and compare the findings with a tooth-coloured compomer. The null hypotheses of the study were as follows: (1) all the colours of both manufacturers would cause higher increase in temperature of the pulp than the tooth-coloured compomer; (2) the same colours of compomers from different manufacturers would produce similar increase in temperature; (3) there would be differences in the increase in temperature during curing of different colours from the same manufacturer; and (4) increase in intrapulpal temperature during light curing of any CCs would not exceed the critical value (5.5 °C) at both 20th and 40th second of irradiation period.

## Materials and Methods

This study was approved by the Human Ethical Committee of the local Medicine Faculty with the reference number 60116787-020/65029 on 25 September 2018.

### Preparation of the specimens

An extracted human mandibular second primary molar tooth was used in the experiments. In order to prevent morphological and structural differences that may occur due to the use of multiple teeth, the study was carried out on a single sample tooth model.

**Figure 1:**
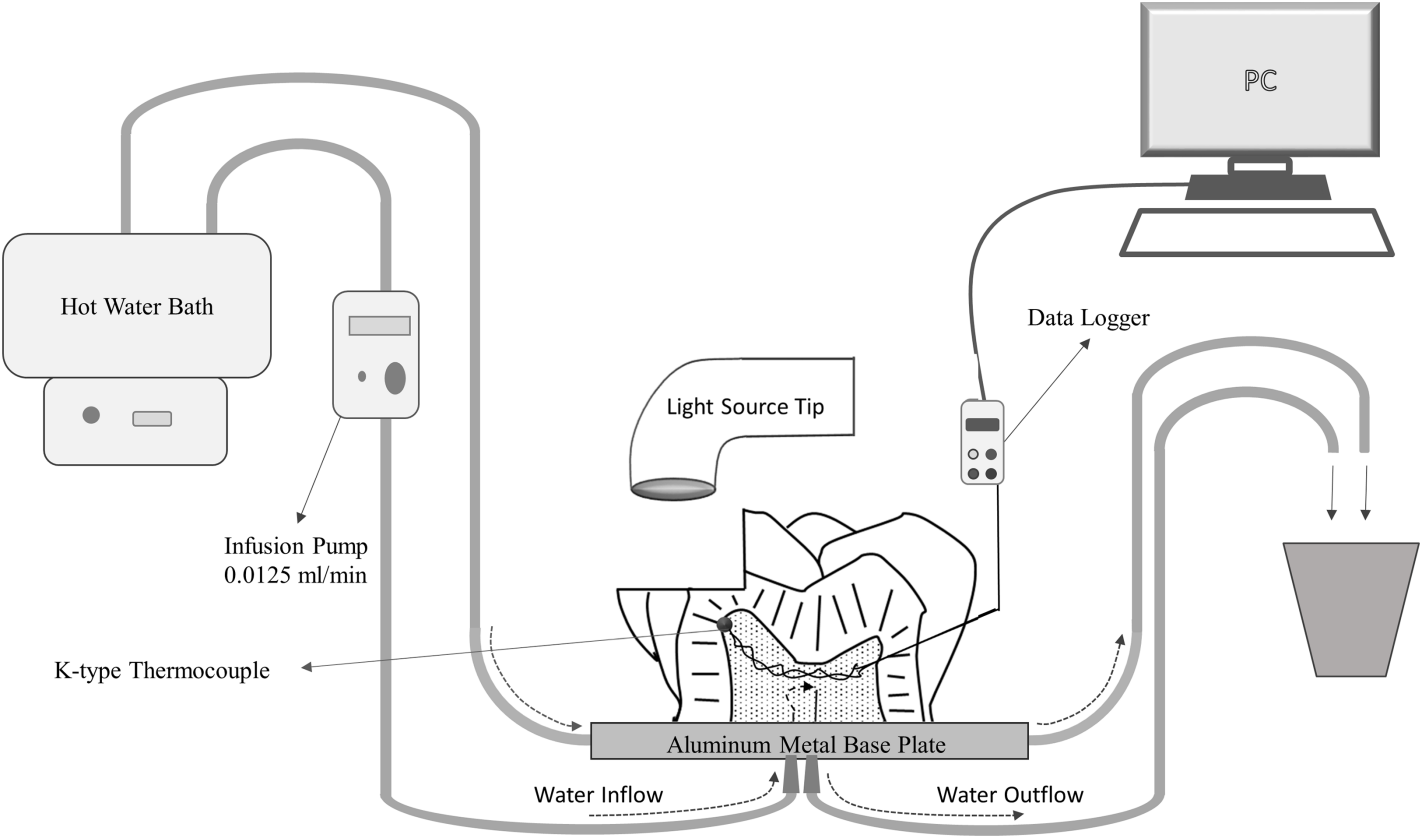
Schematic drawing of the primary second molar tooth and class II cavity with experimental pulp microcirculation model.

A Class II cavity (mesial-occlusal) with depth of two mm, width of three mm and pulpal wall thickness of one mm was prepared on the primary mandibular second molar tooth (Fig. 1). Thickness of pulpal dentin wall was determined with a calliper and radiographic examination. The root was separated approximately one mm below the cementoenamel junction perpendicular to the long axis of the tooth. The remnant pulpal tissues were removed with an excavator and the pulp chamber was irrigated with distilled water and dried with air. In order to insert the thermocouple, the entrance to the pulp chamber was prepared as needed (Fig. 1).

### Study design

A total of 15 groups and seven different CCs: pink, blue, gold, silver, orange, lemon, green, respectively from two different manufacturers (Groups 1–7: Twinky Star; VOCO, Cuxhaven, Germany. Groups 8–14: Nova Rainbow; IMICRYL, Konya, Turkey) and a tooth-coloured compomer (Group 15: Dyract XP, DENTSPLY, Weybridge, UK.) were included in the study.

According to the reference study results by Savas et al. (2014); they had a very large effect size (*F* = 1.388), assuming we can achieve a lower level of effect size (*F* = 0.5) a power analysis was performed before the study. Accordingly, when 90 samples (six samples for each group) were included in the study that would result in 80% power with 95% confidence. We included 10 samples for each group (total 150 samples) in the present study. For temperature increase results, we had a very large effect size (*F* = 1.15) and with this result we reached 100% power with 95% confidence.

A K-type thermocouple (TT-K-30-SLE; Omega Engineering inc, Stanford, CT, USA) was placed in the primary second molar tooth, in contact with the pulp ceiling with thermal grease (ZM-STG2; Zalman Tech Co Ltd, Dongan-gu, South Korea) (Fig. 1). The space around the thermocouple wire was filled with light-curing glass ionomer cement (Ionoseal; Voco, Cuxhaven, Germany) to avoid leakage from the system. The thermocouple cable was connected to a data logger (DT-3891G; CEM, Shenzhen, PRC) that was connected to a computer, through which temperature changes were monitored.

**Figure 2:**
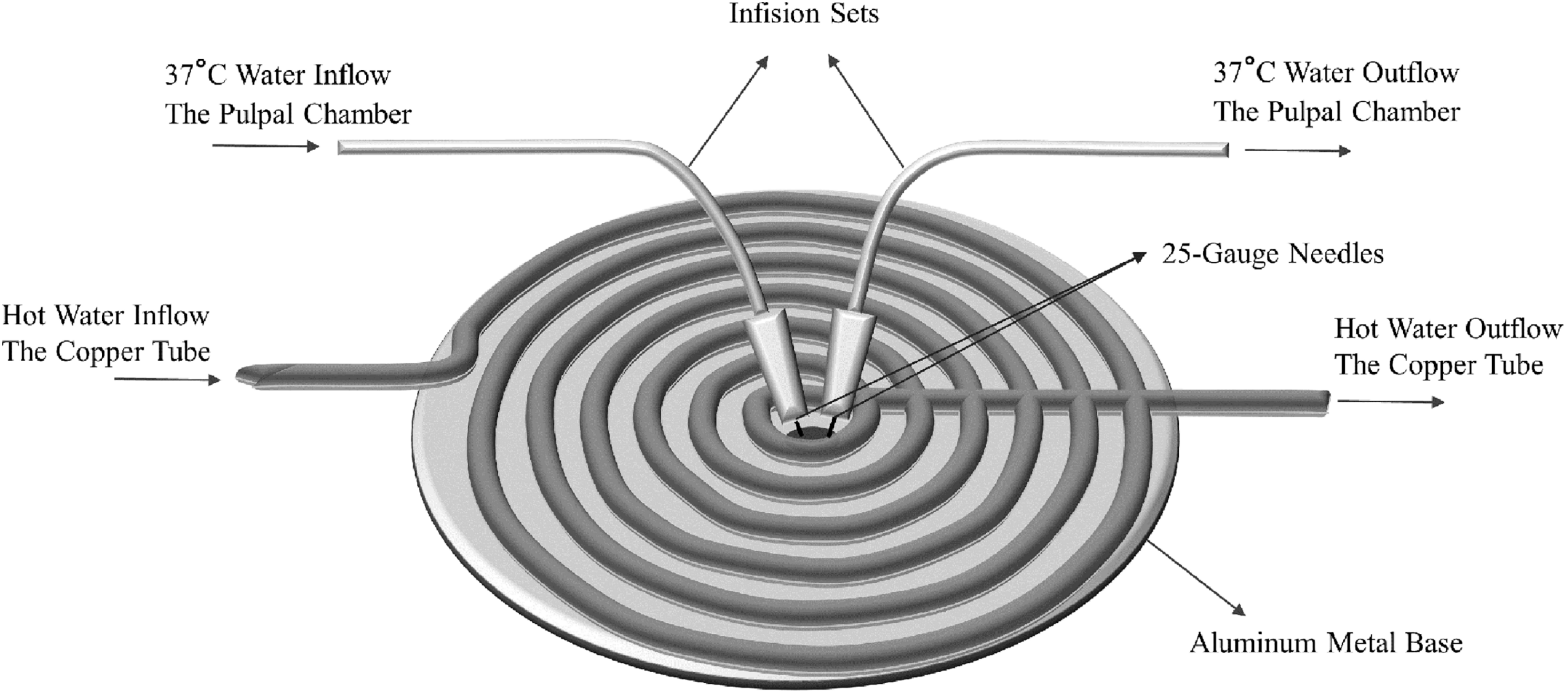
Bottom view of the temperature-controlled aluminium base plate (TCAP), which is part of the experimental apparatus to regulate the tooth physiological temperature.

Pulpal blood microcirculation and temperature regulation of the primary molar tooth within physiological limits (37 ± 1 °C) were performed using an experimental mechanism (Fig. 1). Two 25-gauge needles (8696569000777; Hayat Medical Co., Istanbul, Turkey) were placed to provide intra-chamber microcirculation through the hole of a temperature-controlled aluminium base plate (TCAP) and were used as a channel for inflow and outflow of distilled water. The primary molar tooth was fixed on the TCAP with light-curing glass ionomer cement (Ionoseal; Voco, Cuxhaven, Germany) in such a way that the needles overlapped with the pulp chamber (Fig. 1). The distilled water was passed through the pulp chamber at the rate of 0.0125 ml/min using an infusion pump set (IP12A; Biocare, Shenzhen, PRC) (Fig. 1). In order to maintain physiologic temperature in the pulp chamber, a four mm diameter spiral shaped copper tube was attached under the aluminium base and connected to a water bath with a standard infusion set. Hot water was passed through the copper tube to regulate the physiologic temperature of the TCAP (Fig. 2).

Compomers were applied to Class II cavity and all were polymerised with the same light curing unit (Demi Plus; Kerr, Middleton, WI, USA, 1,200 mW/cm^2^). The CC materials were polymerised for 40 s according to the manufacturer’s instructions. However, for the purpose of the experimental research, both 20th and 40th second Δ*t* measurements were recorded. As per the manufacturer’s instructions, the Dyract XP tooth-coloured compomer used in Group 15 required an irradiation time of 10 s, in this group 10th, 20th and 40th second Δ*t* measurements were recorded. The cavity was not coated with adhesive material before application of the compomers, as the preparation was done on a single tooth model. Thus, after each measurement, the compomer material was easily removed from the cavity with a probe.

Intra-pulpal temperature changes were evaluated while implementing curing unit in the occlusal direction of the Class II cavity from a distance of one mm, which was achieved with a cover glass of one mm thickness. All measurements were made at 37 ± 1 °C and only during the light-curing process of the compomers. The differences between initial and maximum temperatures measured in the pulp chamber during curing of compomers were recorded and the Δ*t* values obtained in all groups were compared.

### Statistical analyses

The statistical analyses were performed by using the IBM SPSS Software (SPSS v23.0; SPSS Inc., Chicago, IL, USA). The Shapiro–Wilk omnibus normality test, Kruskal–Wallis test followed by Mann–Whitney *U* multiple comparisons test were used to analyse the differences in temperature changes between the groups at a significance level of *p* < 0.05.

## Results

**Figure 3:**
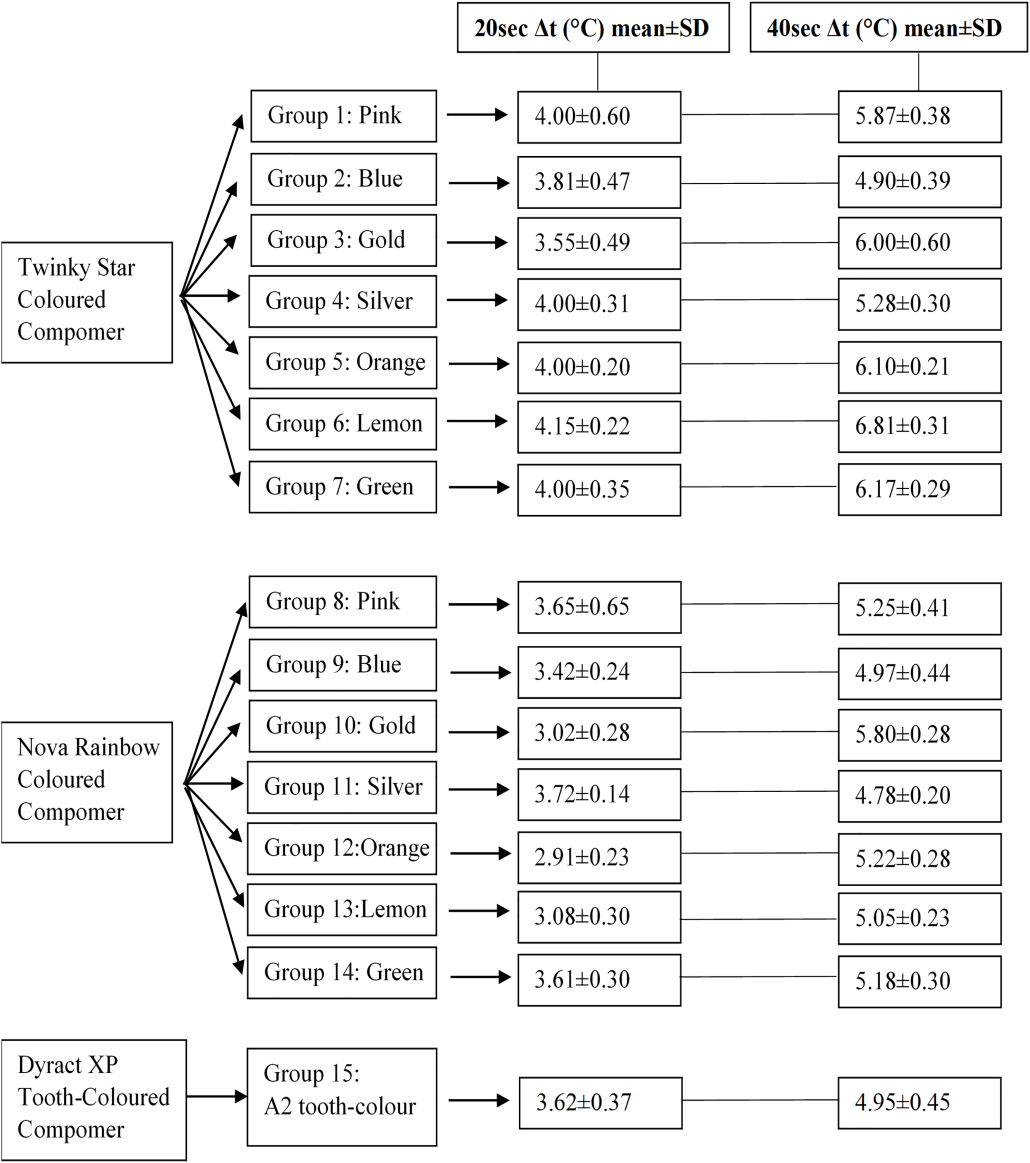
Study groups, tested compomer materials and mean Δ*t* values of the groups at the end of 20 and 40 s.

The mean and standard deviation values of temperature increase obtained in all groups according to the colours and irradiation times are shown in Fig. 3. The statistical differences between the different colours of the same manufacturer, between the same colours of different manufacturers and comparisons of the CCs and tooth-coloured compomer are summarised in Tables 1, 2 and 3.

**Table 1:** Differences between Δ*t* values of coloured compomers from two different manufacturers.

Colours	Twinky star 40 s Δ*t* (°C) mean ± SD	Nova rainbow 40 s Δ*t* (°C) mean ± SD	*p*-value
Pink	5.87 ± 0.38	5.25 ± 0.41	>0.05
Blue	4.90 ± 0.39	4.97 ± 0.44	>0.05
Gold	6.00 ± 0.60	5.80 ± 0.28	>0.05
Silver	5.28 ± 0.30	4.78 ± 0.20	>0.05
Orange	6.10 ± 0.21	5.22 ± 0.28	0.0001
Lemon	6.81 ± 0.31	5.05 ± 0.23	0.0001
Green	6.17 ± 0.29	5.18 ± 0.30	0.0001

**Notes:**

Δ*t*, temperature change in pulp chamber; SD, Standard deviation.

*p* ≤ 0.05 means statistically significant difference.

**Table 2:** Differences between Δ*t* values of Twinky star CCs and Dyract XP tooth-coloured compomer.

Colours	Twinky star CCs 40 s Δ*t* (°C) mean ± SD	Dyract XP tooth-coloured compomer 40 s Δ*t* (°C) mean ± SD	*p*-value
Pink	5.87 ± 0.38^abc^**		0.0001
Blue	4.90 ± 0.39^e^*		>0.05
Gold	6.00 ± 0.60^bc^**		0.0001
Silver	5.28 ± 0.30^ae^*		>0.05
Orange	6.10 ± 0.21^c^**		0.0001
Lemon	6.81 ± 0.31^d^**		0.0001
Green	6.17 ± 0.29^cd^**		0.0001
Tooth colour-A2		4.95 ± 0.45*	

**Notes:**

Δ*t*, temperature change in pulp chamber; SD, Standard deviation; CCs, coloured compomers.

*p* ≤ 0.05 means statistically significant difference. The values indicated by different superscript small letters show statistical significant difference. The values indicated by * and ** are statistical significantly different from each other.

**Table 3:** Differences between Δ*t* values of nova rainbow CCs and Dyract XP tooth-coloured compomer.

Colours	Nova rainbow CCs 40 s Δ*t* (°C) mean ± SD	Dyract XP tooth-coloured compomer 40 s Δ*t* (°C) mean ± SD	*p*-value
Pink	5.25 ± 0.41^ab^*		≥0.05
Blue	4.97 ± 0.44^a^*		≥0.05
Gold	5.80 ± 0.28^b^**		0.0001
Silver	4.78 ± 0.20^ac^*		≥0.05
Orange	5.22 ± 0.28^ab^*		≥0.05
Lemon	5.05 ± 0.23^ac^*		≥0.05
Green	5.18 ± 0.30^ab^*		≥0.05
Tooth colour A2		4.95 ± 0.45*	

**Notes:**

Δ*t*, temperature change in pulp chamber; SD, Standard deviation; CCs, coloured compomers.

*p* ≤ 0.05 means statistically significant difference. The values indicated by different superscript small letters show statistical significant difference. The values indicated by * and ** are statistical significantly different from each other.

In all groups, the measured Δ*t* values at the 20th second were significantly lower than the values measured at the 40th second (*p* = 0.0001). At the end of 20 s, Δ*t* values of the orange and lemon shades from Twinky Star were statistically significantly higher than the same colours of Nova Rainbow (*p* = 0.0001).

At the end of 40-s irradiation period, the orange, lemon and green colours of Nova Rainbow resulted in significantly lower Δ*t* values than the same colours of Twinky Star (*p* = 0.0001). The silver, blue, lemon, green, orange and pink colours of Nova Rainbow and the blue and silver shades of Twinky Star demonstrated lower Δ*t* values than the reported critical temperature increase (5.5 °C) (Zach & Cohen, 1965).

In Group 15, the mean increase in pulpal temperature at the end of the irradiation period of 40 swas statistical significantly lower than in the Groups 1, 3, 5, 6, 7 and 10. Only the mean Δ*t* values of the Group 2 and Group 11 were lower than the Group 15 but the differences were not statistically significant (Tables 2 and 3).

Among all measurements, the lowest mean increase in temperature of the pulp chamber was observed in the orange colour of Nova Rainbow at the 20th second of the irradiation time and this value (2.91 ± 0.23 °C) was even significantly lower than the measured mean Δ*t* value (3.34 ± 0.19 °C) at the 10th second of the irradiation period in Group 15 (*p* = 0.0001).

## Discussion

Although, maintaining the vitality of the pulp is one of the primary objectives of restorative treatments in both primary and permanent dentitions, many physical, chemical, and thermal factors can affect the health of the pulp tissue during restorative procedures (Langeland, 1961). For many years, several studies have investigated the thermal effects of light-curing process on pulp tissues (Zach & Cohen, 1965; Lloyd, Joshi & McGlynn, 1986; Hannig & Bott, 1999; Baroudi, Silikas & Watts, 2009). However, the threshold of temperature increase that can be tolerated by the pulp tissue remains unknown in both primary and permanent dentitions. A study by Zach & Cohen (1965) in rhesus monkeys showed that 15% of the teeth developed necrosis when the healthy pulp was exposed to an increase in temperature of only 5.5 °C (Zach & Cohen, 1965). Similarly, the results of a study conducted by Pohto & Scheinin (1958) indicated that the critical temperature for irreversible damage to the pulp begins at 42–42.5 °C (Pohto & Scheinin, 1958). In another previous study performed on human teeth, it was found that short-time temperature increases in the range of 8.9–14.7 °C did not damage the pulp tissue (Baldissara, Catapano & Scotti, 1997). Safe temperature value for pulp tissue can give more accurate results on culture cells with different time applications (Uhl, Volpel & Sigusch, 2006; Schmalz et al., 1999). Although the value of the critical temperature rise that causes pulp damage in primary teeth remains unknown, it should be accepted that the increase in intra-pulpal temperature during curing of restorative materials should be as low as possible.

Pulpal microcirculation plays a crucial role against temperature changes when the tooth is affected by a thermal stimulus. When pulpal temperature exceeds 43 °C, the neurons in the pulp are stimulated and blood circulation to the pulp chamber increases, which may be considered as a mechanism for dissipation of heat (Raab, 1992). The role of pulpal microcirculation in primary dentition as a cooling agent during dental procedures has not been extensively studied in the literature. In most in vitro studies, the teeth are placed in a water tank containing standing water at 37 °C (Pohto & Scheinin, 1958; Yazici et al., 2006; Mank, Steineck & Brauchli, 2011; Oberholzer et al., 2012). In the present study, in order to simulate the circulation of the pulp chamber, a pulpal circulation mechanism was created similar to the model used in previous studies (Savas et al., 2014; Ramoglu et al., 2015; Yaşa et al., 2017). This mechanism allowed water to circulate within the pulp chamber at a defined flow rate and pressure to simulate in vivo conditions. In the present study, in addition to the mechanism used in the past studies, a copper tube attached under aluminium base and connected to hot water bath was used in order to maintain physiological temperature (37 ± 1 °C) of the experimental setup. These mechanisms provided more realistic results, because simulation of pulpal microcirculation reflected clinical conditions better than experiments without water flow.

The present study was carried out on one sample primary tooth for all experiments without any change in the size of the cavity to eliminate the anatomical and structural differences that may occur with the use of multiple extracted human teeth. It was reported in a previous study that there was no significant difference in the pulpal temperature rise with and without the application of a dentin-bonding agent (Hannig & Bott, 1999). Our preliminary results also showed no statistically significant differences between the temperature changes with and without dentin bonding. Therefore, compomer restorations were filled in the cavity prepared on the primary second molar tooth without application of any dentine adhesive agent and so it was possible to replace the polymerised compomer material after repeated measurements.

At the end of 40-s irradiation period, although the differences were not statistically significant, the blue shade of Twinky Star and the silver colour of Nova Rainbow showed lower Δ*t* values than the tooth-coloured compomer. Therefore, the null hypothesis that all the CCs from both manufacturers would cause higher increases in temperature of the pulp than the tooth-coloured materials, was not confirmed.

According to the manufacturer, the difference between Twinky Star and tooth-coloured compomers are the added pigments while the other components are similar in both materials (Hwang et al., 2007; Khodadadi, Khafri & Aziznezhad, 2016). Therefore, differences in temperature changes could be attributed to the type and amount of the added pigments. Furthermore, several previous studies had shown that a change in exotherm that causes the temperature increase in pulp tissue was because of differences in the materials’ composition (Lloyd, Joshi & McGlynn, 1986; Adamson, Lloyd & Watts, 1988; Al-Qudah et al., 2005). Baroudi, Silikas & Watts (2009) concluded that flowable composites exhibited higher temperature rises than non-flowable composites, and they attributed this result to flowable composites’ lower filler loading and higher resin content, which should increase the exothermic reaction and according to them the highly exothermic nature of the setting reaction of flowable composite produced substantial temperature rise. Although there are not enough data about the mechanism that cause different temperature increases in the pulp during light curing of different CCs, possibly it is because the different exothermic reaction of different amount of resin content of the materials. Another remarkable result of this study is that the silver colour of Nova Rainbow (Group-11) showed the highest temperature increase in 20th second and the lowest at the end of 40 s among all the materials examined. Since the same light curing device is applied to the materials for the same period in all groups, this finding is thought to be due to the fact that the exothermic reaction of the resin component of this compomer material occurs faster than the others in the first 20 s and then slows down.

At the end of the irradiation period of 40 s, the silver, blue, lemon, green, orange and pink colours of Nova Rainbow and the blue and silver shades of Twinky Star, which demonstrated lower Δ*t* values than the reported critical temperature increase (5.5 °C) (Zach & Cohen, 1965; Pohto & Scheinin, 1958) could be considered as reliable restorative materials. Most of the Twinky Star CCs caused an increase in intra-pulpal temperature of more than 6 °C, therefore, especially the orange, lemon or green colours of Nova Rainbow, which resulted in statistically significant lower increase in temperature than the same colours of Twinky Star, may be the choice of restorative material. Furthermore with this result the second null hypothesis of the study that the same colours from different manufacturers would produce similar increase in intrapulpal temperature is rejected.

As a result of statistical analyses, it was detected that different colours of the same manufacturer caused significantly different intrapulpal temperature increases. For example the lemon shade of Twinky Star and the gold colour of Nova rainbow showed significantly higher Δ*t* values than most of the other colours of the same manufacturer (Tables 2 and 3). Thus the third null hypothesis of the study is confirmed.

To the best of our knowledge, no study has investigated the increases in intrapulpal temperature during polymerisation of CCs in primary teeth. In previous studies, some properties of CCs, such as coefficient of heat conductivity, conversion degree, curing depth and surface microhardness have been investigated; however, no clear opinions or consensus have been reported (Hwang et al., 2007; Vandenbulcke et al., 2010; Guler et al., 2017; Atabak & Bodur, 2011; Jafari, Javadinejad & Mirzakochaki, 2015). One study reported that, the silver shade of Twinky Star showed significantly higher coefficient of heat conductivity than other colours and should not be used in restoration of deep cavities (Guler et al., 2017). On the contrary, the results of the present study showed that the Twinky Star silver shade did not reach the critical temperature of 5.5 °C and can be used safely in the restoration of deep cavities.

The blue shade of Twinky Star has been reported to demonstrate higher curing depth and conversion degree than the other colours (Vandenbulcke et al., 2010; Atabak & Bodur, 2011). It was stated that this property of the compomer could be related to the type, size and amounts of glittering components in the material (Atabak & Bodur, 2011). However, in the present study, the Twinky Star blue shade was found to cause a lower increase in intra-pulpal temperature compared to the other colours from the same manufacturer.

Jafari, Javadinejad & Mirzakochaki (2015) reported that pigments in darker CCs such as shade Blue absorb more light, thus reducing the depth of penetration of the light into the resin material (Jafari, Javadinejad & Mirzakochaki, 2015). In the present study, the Twinky Star blue shade caused lower increase in temperature of the pulp chamber compared to the other compomers. This may be attributed to the fact that the pigments of the shade Blue absorb more light and prevent the penetration of light into the pulp chamber, in accordance with that reported in the study by Jafari et al. Similarly, the blue colour of Nova Rainbow was one of the colours that caused the least increase in pulpal temperature. However, unlike the result reported in the study by Jafari, Javadinejad & Mirzakochaki (2015), the silver shade, which is a light colour, was found to cause the lowest increase in temperature of the pulp chamber. On the other hand, Hwang et al. (2007), reported that the Twinky Star blue and silver shades had high light transmission property; therefore, allowed deeper penetration of light without being absorbed. The study also reported that the shade Gold demonstrated low light transmission; therefore, did not allow deep penetration of light (Hwang et al., 2007). However, the findings of the present study are not in consensus with that reported in the above-mentioned study. In the present study, the Twinky Star blue and silver colours transmitted less light energy to the pulp, hence resulted in lower increase in intra-pulpal temperature. In addition, intra-pulpal temperature increase (6 °C) during curing of the gold shade was found to be above the critical value and was concluded that higher penetration of light occurred into the pulp chamber.

As a null hypothesis of the study it was thought that any CCs would not exceed the reported critical value (5.5 °C) at both 20th and 40th second of the irradiation period. For the 20th second values the null hypothesis is confirmed but at the end of 40 s it is rejected. Because almost half of the tested CC materials had shown higher Δ*t* values than 5.5 °C.

## Conclusions

The temperature increases in primary tooth pulp during light-curing of CCs showed significant differences according to the shade of the material and the manufacturer. It was observed that CC materials may cause temperature increase in pulp chamber more than the critical value at the end of 40-s irradiation; furthermore, an increase in the irradiation time significantly led to an increase in the intrapulpal temperature during light curing of both two different CCs. Therefore, the present study suggests that manufacturers and firms should focus on production of new CC materials with shorter irradiation time for polymerisation in order to protect pulp tissue from thermal damages.

## Supplemental Information

10.7717/peerj.7284/supp-1 Supplemental Information 1 Raw data of temperature change values measured in the experiments. Each data indicates mean intrapulpal temperature increases measured during light curing process of different coloured compomers in Groups 1–2 and tooth coloured compomers in Group 3.

10.7717/peerj.7284/supp-2 Supplemental Information 2 Shapiro–Wilk test results of the groups included in the study.

## Additional Information and Declarations

### Data Availability

The following information was supplied regarding data availability: The raw measurements are available in Datasets S1 and S2. Dataset S1 shows all temperature change values measured in study groups and the statistical differences among the groups. Dataset S2 shows the results of Shapiro–Wilk test.
